# Supplementary material for: Practice beyond performance stabilization increases the use of online adjustments to unpredictable perturbations in an interceptive task
Source: PLoS One. 2025 Nov 5;20(11):e0313665. doi: 10.1371/journal.pone.0313665 (PMC12588517; doi:10.1371/journal.pone.0313665)
Supplement: Attachment — Submitted filename: Response to reviewers.docx [file pone.0313665.s003.docx]

We are grateful to the reviewers for their suggestions and the opportunity to improve the manuscript to make it clearer in this version. The comments made us think and write a lot about them, so some answers are quite long, and the whole file is nearly a new manuscript. Sorry about that. However, the manuscript improved significantly in our analysis. Thank you again about your comments. All changes in the manuscript are shown in blue.

**REVIEWER 1**

Major comments:

The first comment involves many concepts and method. So, to provide a clearer response, we have divided comment 1 into subcomments: 1a, 1b, 1c, 1d, 1e, 1f, and 1g.

Comment 1a) As pointed out above, the study focuses on the stabilization process as a consequence of motor practice. But the characterization of the level of stabilization of both groups is not clearly shown. How did performance changed (or improved) from the achievement of intercepting the target three trials in a row to the achievement of intercepting the target three trials in a row six times (respectively, for stabilization and superstabilization groups)?

Answer: This is our study's central point and we appreciate your comment. We have now worked on clarifying it (lines 70-79). Performance is considered stable when errors remain within a predefined range of accuracy defined as acceptable

for a given task, and it is task-dependent. In our study, after conducting many pilot studies, we identified that volunteers were unlikely to repeat a performance with an error smaller than -5 cm to +5 cm from the center of the target more than three trials in a row over 500 attempts. Therefore, we adopted the criterion for this task as three consecutive trials with an error between -5 cm and +5 cm. This same criterion has been used in previous studies (Couto et al., 2021; Campos et al., 2022) for this task.

Specifically considering the two levels of stabilization, for performance to be stable (i.e., consistently reproduced within a small margin of error), there must be a spatiotemporal standardization of the movement, resulting in correct responses in consecutive trials, which allows us to consider that performance stabilization has been achieved (Burdet et al., 2006; Corrêa et al., 2015; Loschiavo-Alvares et al. 2023). This rationale has been applied in studies involving complex coincident-timing tasks, in which performance stabilization is reached when the error is equal to or less than 30 ms, and this level of performance is repeated for three consecutive trials (Fonseca et al., 2012; Ugrinowitsh et al., 2011; Campos et al., 2022; Loschiavo-Alvares et al., 2023). In an isometric force control task, we also ran pilot studies and found that participant could perform no more than four consecutive trials with an error smaller than 5% of the task goal (i.e., reach 40% of the maximum force). So, we adopted this performance criterion in a force control task (Santos et al., 2017). As described above, all these criteria were established following pilot studies to identify how volunteers perform according to the task.

Considering superstabilization requires another criterion, but pilot studies have shown that due to task constraints, performance accuracy does not increase indefinitely, as this would require a level of expertise. We would never be able to perform this study if we consider expertise as 10 years of practice or 10 thousand trials (Ericsson & Smith, 1994). Therefore, superstabilization was operationally defined as repeatedly maintaining stabilized performance. Again, based on pilot studies, we have adopted repeating the stabilization criteria for six blocks, as adopted in previous studies (Fonseca et al., 2012; Ugrinowitsch et al., 2011; Corrêa et al., 2015; Couto et al., 2021; Campos et al., 2022).

Previous studies (Ugrinowitsch et al., 2014 and Couto et al., 2021) have shown that, despite statistically equal accuracy during the learning phase, greater performance variability is identified when reaching superstabilization compared to those who practiced until stabilization. Still, most other studies that compared performance in stabilization with superstabilization did not find this behavior. This may happen because the differences can be found in control measures not in performance, and it depend on the characteristics of the task as well. However, this discussion about what happens in the phase prior to the insertion of perturbations was not the aim of the present study but rather to investigate behavior in the face of unpredictable perturbations.

Given the established criterion, we did not expect to find differences in performance during the first phase of the study (i.e., learning phase) but rather when participants had to face perturbations. Indeed, studies have shown that under such conditions, groups that practiced until reaching the superstabilization performance criterion (i.e., six blocks of three or four consecutive successful trials) could better deal with the perturbations than those that practiced only until reaching the performance criterion for what we named stabilization (Fonseca et al., 2012; Ugrinowitsch et al., 2014; Corrêa et al., 2015; Santos et al., 2017).

Comment 1b) Comparing the groups, how was variability reduced to allow performance to be considered super stable?

Answer: Stability can be interpreted in different ways. In the present study, we are not interpreting stability only as associated with reduced variability, but also concerning increased accuracy. More specifically, the definition of different stabilization levels is based on absolute spatial error within a quite small error range (accuracy), range repeated a few times (consistency). A previous study conducted by our group (Couto et al., 2021) with this same task shows that both the variability of performance and control measures, of the levels of superstabilization and stabilization, decreased throughout the learning phase, even with the superstabilization group showing greater variability of performance than the stabilization group. Here is important to highlight that the criterion adopted to indicate stability is highly stringent. It often makes it impossible to identify a difference between stabilization and superstabilization during the learning phase. Once more, maybe we could find differences between stabilization level and expertise, but it is a question for another study.

Comment: 1c) Authors do not report performance scores for each group other than the variables number of corrections, time to peak velocity, and constant error with respect to Perturbation I (Fig. 3) and Perturbation II (Fig. 4). Interestingly, the values of constant error of both groups for PrePI1 (Fig. 3c) and PrePII1 (Fig. 4c), which are the first information available since the separation of groups, do seem very similar. It is necessary to add explicit data on the differences of the interceptive task performance between the two groups.

Answer: As we understand it, this comment refers to the differences resulting from the manipulation of the two levels of stabilization, so we will proceed accordingly.

Considering that both stabilization levels have the same performance criterion to stop practicing, both are expected to have similar performance, and the difference in task performance will only appear when the perturbation is introduced and/or after its removal. Before the perturbation, the only distinction between groups that achieve stabilization and superstabilization is in performance variability, as demonstrated in some previous studies (Ugrinowitsch et al., 2014; Couto et al., 2021).

Furthermore, the expected explicit difference between two levels of stabilization in the first phase could appear when comparing an expert with someone who has merely stabilized the performance, which is not the case in the present study. As previously mentioned, the stabilization levels may not have a difference in performance accuracy in the learning phase since the practice ends when participants reach the criterion established for the task, which is the same for both groups (i.e., three trials in a row with AE < + 5cm). Interestingly, the differences appear when they need to deal with perturbations. Probably this difference is because by prolonging the practice beyond stabilization (i.e., superstabilization), when participants have more opportunities to obtain information about the target's displacement speed and the interception location, and thus improve their skill in the task, observed when facing perturbations. For example, when the perturbation was inserted, Fonseca et al. (2012) and Ugrinowitsch et al. (2014) found a difference in the organization of the components of the complex task, and Santos et al. (2017) found a difference in co-contraction, although none of the studies showed difference between the two levels of stabilization during the learning phase. It means that superstabilization allowed for more information about the task and its execution, facilitating adjustments in the control mechanisms and achieving the same performance. These results show the importance of continuing to investigate the difference in stabilization levels that allow for better performance or better control when facing perturbations.

In this study, we adopted performance and control mechanism measures. Like some previous studies, the PRE P_fast_ (in the first version named PI) data did not show any difference between the stabilization and superstabilization levels in the

First phase (we explained it above). For example, the constant error increased when facing the perturbation and return to the level of Pre-Perturbation in the control trial – PosP (speed used in the learning phase).

Considering the no differences between groups during PreP, one explanation is the performance criterion adopted to finish the first phase: the error should be < + 5 cm. As we explained before, this error criterion was adopted based on pilot studies, and it seems correct because the difference between stabilization and superstabilization can only be detected under unpredictable conditions. That is the reason we investigated this question in the manuscript. If we had chosen another error criterion to stop practicing, e.g., ± 20 cm, there would probably be differences in PreP that would make it unfeasible to investigate the effects of levels of stabilization during unpredictable perturbations.

1d) Also, providing details on the amount of practice involved in the preparation phase for both groups are necessary.

Answer: The focus of this study is on the second phase and not on the first. Previous studies (e.g., Couto et al., 2021 and Campos et al., 2022) have already demonstrated that achieving stabilization and superstabilization of performance in the same task and under the same practice conditions as our study ensures the achievement of two different levels of sensorimotor system functionality — that is, two different levels of performance stabilization. This serves as the starting point of the present study, and not the process of reaching the different levels.

This is an important comment because it differentiates our study from many others related to motor learning. Most of the studies adopt a specific number of trials during the learning phase (McGarity-Shipley et al., 2023; Cruz et al., 2024). This procedure gives good control over the amount of practice but not the level of learning. It means that some participants can have different levels of learning in the task, which can influence the results. Differently, some studies use a performance criterion in the learning phase (Santos et al., 2017; Campos et al., 2022). This procedure gives good control over the level of learning because all participants stop practicing at a similar level of performance. Consequently, it gives better control over the effects caused by the variable tested (e.g., feedback, attentional focus) because any difference observed after practicing is caused by the independent variable and not from differences in the level of learning in the task.

Apart from that, we organize data related to the number of trials to reach the criterion of performance for both levels of stabilization. The figure below illustrates the average number of trials achieved by both groups (gray bar: SG and black bar: SSG). The test *t* revealed that the SSG performed more trials than SG *t*(38) = -3.967, p=0.0003. However, we emphasize once again that the focus of this study is on the second phase, when perturbations were inserted, and not on the first.

Comment: 1e) Additionally, the criterion “intercepting the target three trials in a row six times” is not sufficiently clear. Provide details on what exactly “six times” mean in terms of the circumstances of the proposed method; it does not seem to be 18 times in a row (lines 194-195_Now lines201-202).

Answer: Perfect. We do agree with the comment, and have modified the writing on line 202 to make it clearer for the reader. In this new version, you will find: “The Superstabilization Group practiced until they reached the same performance criterion (three right trials in a row), which should be repeated six times throughout the phase. So, Stabilization should perform one block of three right trials in a row, and Superstabilization should perform the same criterion (i.e., three correct trials in a row) for six times”

Comment: 1f) Also, there is an additional definition of criteria for separating the groups (see lines 197-201_Now lines 204-208), stating that an error smaller than or equal 5 cm in 200 trials for the stabilization group and an error smaller than or equal 5 cm in 320 trials for the superstabilization group. How both criteria for separating the groups were simultaneously applied?

Answer: In fact, the criteria was not applied simultaneously. In lines 195-197 cite those participants were randomly assigned into two groups, SG and SSG. Lines 199-203 report the criterion for each group and the number of trials allowed to reach the criterion. SG had 200 trials because this group should perform the criteria only once. The SSG had 320 trials because it should repeat the criteria six times. In fact, participants spent more time learning the VIT and reaching the performance criteria. That is the reason of 200 trials. The SSG should repeat the performance criteria for six times, it was not necessary to improve the amount of practice by six. All these numbers were identified in pilot studies. Participants that did not reach the performance criteria were excluded from the experiment, as happened with two participants (description between lines 216 and 217).

We have revised the section (lines 208–213) to facilitate the reader’s understanding.

Comment: 1g) A third criterion was also used (movement time between 200-250 ms); although the use of feedback is described for different time ranges, what would happen to trials out of the expected duration (200-250 ms)? Overall, this description is very confusing. Please, clarify this issue.

Thank you for the comment. We have revised it (lines 218–223) for clarity. Below there is the explanation concerning the movement time control.

In this study, the movement time does not refer to an independent variable but rather a control variable. We aimed to identify the strategies used and, from them, infer the control mechanism when groups that achieved two different levels of performance stabilization were exposed to perturbations requiring modifications of the prior planning after movement onset.

In interception tasks involving moving targets, movements lasting 150 milliseconds are considered fast (Tresilian, 1995). However, restricting movement time to 150 milliseconds would not allow us to identify control strategies that rely on sensory feedback to adjust planning before perturbations and, consequently, the use of the two possible mechanisms of control. Therefore, to determine whether participants predominantly used pre-programmed control or online feedback when facing perturbations inserted immediately after movement onset — within a short time window for corrections—they were required to learn a movement time ranging between 200 and 250 milliseconds.

Once more, the procedures were determined through pilot studies. During pilot studies, when movement time was not controlled, we observed that participants executed movements slowly, exceeding 300 milliseconds, and made multiple corrections along the trajectory. This altered the nature of the ballistic movement, which was the focus of this study. Consequently, we didn’t observe participants controlling the movement using predominantly pre-programmed mechanisms. So, we needed a time constraint in the task to find participants using both control mechanisms. After testing different time constraints, it was identified that 200-250 give us the possibility of investigating the control mechanisms in the task.

Throughout the experiment, if any participant did not perform the movement between 200 and 250 ms, they were instructed to adjust it in the next trial, which they successfully did. The number of trials and the feedback provided were sufficient for all participants to learn to perform the movements within the established movement time while learning the task, as shown in the figure below.

Comment: 2) In a similar vein as above, there is a central question regarding the proposed design. If the main interest of authors was to understand the effects of practice on the performance of an interceptive task when target velocity changes throughout its arrival time, why did not participants practice (during preparation or acquisition phase of the study) in the same conditions (with change in target velocity) instead of practicing under target constant velocity? So, the criteria for defining both groups would be specific for changes in target velocity, the proposed goal of the study. Authors need to justify the adequacy of such a design and the consequences of their choices; authors did not mention this aspect in the Introduction and/or in the Discussion section.

Answer: This comment is interesting and has a good suggestion. However, the suggestion has the variable practice as an independent variable, and in our study, it is the level of stabilization. So, it is another interesting project. In order to make it clear that the state-of-the-art considering studies investigating motor performance and control in response to unpredictable perturbations lies in constant practice. So, we used constant practice and manipulated the two levels of stabilization. Apart of that, we revised the introduction and discussion to make it clearer, as described below*. Our study takes a step forward compared to previous research by focusing on this issue in a ballistic task with only one degree of freedom.

*****We have added the following to the introduction:

1) “under constant schedule practice” – lines 69 and 70.

2) “Over a constant schedule” – line 76.

***** We also have modified the following sentence:

“The practice of an interception task in predictable contexts favors the use of the pre-programming mechanism” to “When the practice of an interception task is in a predictable context, under constant practice schedule, the pre-programming mechanism predominates” – Lines 107-108.

*****We have added the following to the discussion:

1) even though superstabilization was achieved under constant schedule practice, as previous studies [8, 13] – lines 443 and 444.

In our study, we did not base our approach on the theoretical assumptions of varied practice (Moxley, 1979) or practice schedules (Shea & Morgan, 1979).

In this study, we assume that extending practice beyond performance stabilization allows the sensorimotor system to continue acquiring information about the task. Consequently, the internal representation, named internal model (line 79) formed during the stabilization process gains information when practice extends beyond stabilization (i.e., superstabilization), even under constant practice schedule. This higher amount of information related to the task can be used when facing predictable perturbations (Ugrinowitsch et al., 2014) or unpredictable, as in complex coincident timing tasks (e.g., Fonseca et al., 2012) and force control tasks (Santos et al., 2017).

In general, the ability of changing of the internal model comes from the different strategies tested by the sensorimotor system for reaching superstabilization. In our study, we assume that the process of exploring different solution strategies for the motor problem (intercepting the target) while reaching superstabilization not only enhances the ability of the internal model to correct the first command but also facilitates the training of sensory and internal feedback circuits (Desmurget & Grafton, 2010), which are essential for correcting ballistic movements.

Considering that our study focused on the hypothesis that practice beyond stabilizing performance provides learners with the opportunity to test different control strategies to achieve the task goal in consecutive trials—and that it is this process of testing strategies that grants ability of changing to the internal model and strengthens sensory and internal feedback circuits—a logical first step in testing our question was through constant practice schedule.

Now that we have established that achieving superstabilization of performance enhances the ability of changing of the internal model to handle slow perturbations in interception tasks with moving targets, we are now exploring whether manipulating the schedule of practice could promote the flexibility needed to also manage fast perturbations. This investigation is currently underway in our laboratory.

Comment 3) Lines 62-66 (Introduction): “Predictable perturbations are identified before the movement onset; consequently, the action planning may contain the changes necessary to adapt to the perturbation. Conversely, unpredictable perturbations are identified only after the onset of movement; consequently, the action planning has to be reorganized after its triggering to adapt to the perturbation”. I believe that there is a misunderstanding on the distinction between motor action and visual stimuli. Stimuli may be predictable or unpredictable while stimuli perception may occur prior or after movement onset; time constraints involved in motor action and the time stimuli are available to the perceiver may vary. The suggested links between predictable – before onset and unpredictable – after onset do not seem appropriate. Please, consider to rephrase these sentences, making the concepts adequate.

Answer: Thank you for the suggestion. We have revised the sentence in the manuscript (lines 62–65) and provided the updated version below:

“Predictable perturbations can be anticipated, consequently, the action planning may contain the changes necessary to adapt to the perturbation. Conversely, unpredictable perturbations cannot be anticipated, consequently, the action is modified in response to sensorial feedback during the execution”.

This is a crucial point. In our study, not only was the stimulus itself unpredictable, but so was its perception, as we manipulated the timing and the type of its insertion during the perturbation. Consequently, during the perturbation, the perception could not be anticipated, given that it was altered at different times and speeds in unpredictable ways. We have decided this arrangement through multiple pilot studies. The action was initiated based on a constant and predictable visual stimulus. When the speed remained unchanged (without perturbation), the action proceeded as planned, and the results demonstrated accurate interception. However, when the speed was altered without the performer's knowledge after movement initiation, i.e., the change was unpredictable for the performer, requiring adjust the initiated action. Therefore, it is impossible to perceive the change before the onset of the movement. In fact, the instrument was programmed to implement the change only after the movement of the physical effector was identified.

To provide a clearer response, we have divided question 4 into sub-questions: 4a and 4b.

Comment 4a) Lines 245-253_Now lines 261-279 (Material and Methods): there are various aspects of the exposition phase that are not clear and require some discussion as follows: a first aspect refers to nomenclature. Instead of using numbers to differentiate between increase or decrease of target velocity (authors used PI and PII, respectively) and among early, intermediate, or late exposition (authors used, e.g., PI1, PI2, PI3), the terms themselves could have been used to facilitate understanding.

Answer: Great suggestion regarding the nomenclatures. Thank you. We have accepted it and updated the acronyms in the graphs (see figures 3 and 4), captions, and text from line 261-279. And also, we assumed it in this file with the answers.

Comment 4b) A second aspect refers to the reason of separating data as level of the design. For example, why were three levels of responses for increase/decrease in velocity (early, intermediate, and late) used? The statistical analysis (ANOVA) proposed does not use these three levels as an independent variable of the design; why did these data were collected? Presumably, these three levels were averaged to be entered to the ANOVA. In short, it is not clear what is the importance of early, intermediate, and late exposure in this study. In the same manner, it is not clear why pre/post blocks (both with constant target velocity) were used in the experimental design; although these levels were used as independent variable, there is no explanation for using a block of trials with change in target velocity “isolated” between two (pre and post) blocks of trials with constant target velocity. The rationale of the adopted design needs to be clearly explained to readers.

Answer: The comment is perfect. Thank you. We have modified the text to make it clear to the reader, as follows. In the text, the modification appears between lines 263 and 267.

Previous trials are essential as they serve as a reference for understanding what occurs when the perturbation is introduced. In other words, to assess the effects of the perturbation, it is necessary to have a baseline condition without perturbation for comparison.

Post-trials, on the other hand, provide insight into the group's competence based on its level of performance stabilization. More specifically, they reveal the flexibility of the internal model in restoring performance after being disrupted.

Comment 5) Lines 379-380_Now lines 405-406 (Discussion): “… on adjustments to unpredictable perturbations”. The amount of practice provided by the protocol was in trials with target constant velocity (predictable situation). Performance stabilization criteria for each group was reached in those predictable trials. It is not clear, and should be emphasized and discussed by authors, that stabilization criteria were achieved with practice under predictable situation.

Answer: Thank you for the suggestion. We have added a complementary section

between lines 407 and 412, and we report it below.

Participants were initially trained, in a constant schedule of practice, to reach one of these stabilization levels and then assigned to two groups based on their achievement. Constant practice prior the insertion of perturbations enables the formation of an internal model, sufficiently flexible to deal with unpredictable perturbations. For example, in accordance with our results, Santos et al., (2017) [9] showed that superstabilization performance provides higher competence than stabilization performance to deal with unpredictable perturbation in an isometric force control task.

Comment 6) Line 384_Now line 415 (Discussion): “18 random trials”. These trials were not completely random because there were blocks of 3 trials to accommodate pre and post unpredictable block of trials. Provide details of the experimental design.

Answer: You are correct—the appropriate term is pseudorandom. We have made this change in line 415.

The exposure phase consisted of 129 trials, 18 of which were perturbation trials. Among these, nine were fast (P_fast_) and nine were slow (P_slow_). To ensure unpredictability regarding both the type (P_fast_ or P_slow_) and the timing of onset, these trials were randomly distributed throughout the phase. Prior to conducting this study, multiple pilot studies were carried out to assess whether the arrangement of control and perturbation trials effectively maintained unpredictability. Additionally, the same arrangement was previously employed in the study by Fonseca et al. (2012).

For our analysis, we organized blocks of three trials with Perturbation (P) with their respective pre and post trials. Specifically, the nine pre perturbation fast (PreP_fast_) and nine post perturbation fast (PostP_fast_), as well as, the nine pre perturbation slow (PreP_slow_) and nine post perturbation slow (PostP_slow_) were organized into three blocks, each consisting of three trials as follows: PreP, P, PostP.

We revised the text between lines 267 and 279 to enhance clarity regarding the organization.

Comment 7) Lines 386-388_Now 418 and 419 (Discussion): “Thus confirming our hypothesis about the benefits of superstabilization in enhancing control mechanisms and performance outcomes”. This conclusion can be challenged. The adjustments were in response to unpredictable and predictable (pre and post) target velocities. In fact, the predictable ones represent 2/3 of these data submitted to ANOVA. The superstabilization also affected the predictable trials (main effect of group). In the current experimental design, benefits of superstabilization should be indicated by significant group by blocks interaction. Overall, considering three dependent variables (number of corrections, tPV, CE) and three exposures, nine significant group by blocks interactions could be found. However, for the Perturbation I, only three significant interactions were found; similarly, for the Perturbation II, only four significant interactions were found. Thus, this does not confirm the hypothesis as stated by authors. These aspects should be included in the Discussion section.

Answer: Thank you for your comment. We will start this answer returning to the method. The PRE and Post blocks are control trials, the same used during the learning phase. The unpredictable trials, i.e., unpredictable perturbations for the participants, was used to investigate how both levels of stabilization perform and control the movement. This design and data analyses were adopted to investigate participants perform with the perturbation (comparing PRE x Pert) and how behavior returns in the condition without perturbation (PERT x POST), and analyzed the three blocks together. We expected that SSG would have better performance and could change movement control more easily than SG, and SSG should have both measures in POST similar to PRE.

We modified the text between lines 530 and 533 to clarify that we confirmed the hypothesis presented in the introduction: “We hypothesized that superstabilization would better facilitate motor adjustments in response to unpredictable perturbations.” “In general, the results confirm our hypothesis about the benefits of superstabilization in enhancing control mechanisms and performance outcomes, but specially in P_slow_.”

These results generalization is based on the following results: The Student's t-test demonstrated that, when faced with both fast and slow perturbations, the SSG **performed more adjustments** than the SG, as illustrated in figures 3a and 4ª (see below). Regarding the impact of these adjustments on performance, the two-way ANOVA showed that in the second block (P2_slow_) of the slow perturbation, only the SSG was able to maintain the performance level observed in the preceding trials (Pre P2_slow_). In the second block (P2_slow_) of the slow perturbation, the performance accuracy of the SG diminished. Furthermore, in the third block (P3_slow_) of the slow perturbation, the SSG showed a better performance than the SG.

Comment 8) Lines 478-485_Now 512-528 (Limitations and Future Perspectives): The limitation is not clearly described. Review text.

Answer: We appreciate your observation. Thank you. To clarify the limitations, we have rewritten them between lines 508 and 515.

Comment 9) Line 505 (Conclusion): No previous reference to “forward models”. Introduction and Discussion sections could provide some background on this, if this reference is really needed.

Forward models were mentioned in lines 515 and 521. However, we found it appropriate to replace their reference in the conclusion by internal models, that accommodates the forward models and were more frequently mentioned in the introduction and discussion.

Minor points:

Comment 1) lines 74-75 (now 76-78): “… consistent internal representation of the limbs involved in the task and environmental dynamics…”. The representation should refer to limb movements’ program and/or parameters and not to the limb itself.

Answer: The internal representation referred to in this study corresponds to Internal Models.

Due to the necessity of providing context in the initial paragraphs of the introduction, internal models were not mentioned between lines 74 and 75. However, starting from line 79, we began to refer to the internal representation as an internal model. Consequently, the central representations discussed in this work are internal models, distinct from the motor program and parameters proposed by Schmidt (1975) and subsequent researchers.

Conceptually, Internal Models are internal representations that control movements by transforming sensory information into motor actions.

The initial idea of internal models was proposed by Craik (1943) in The Nature of Explanation, where he suggested that organisms use internal representations of the world to generate movements. In the 1990s, Wolpert, Ghahramani, and Jordan (1995) and Wolpert (1997), refined Craik’s concept, defining Internal Models as predictive representations that simulate the motor system’s dynamics during motor planning, control, and learning. These models operate by integrating information from the environment, the body, and previously executed motor commands.

Thus, Internal Models encode information about the limbs involved in the task and the dynamics of the environment. To clarify this idea, we have added the following phrase in lines 80-81: “with the characteristics of the limbs and environmental dynamics involved in the task”.

Comment 2) line 107_Now line 112: “the successful interception of moving interception…”. Repeated words.

Answer: Thank you for your observation. We have made the correction on line 112: “...the successful interception of moving target demands great…”.

Comment 3) lines 113-114_Now line 118: “…the onset of the onset…”. Repeated words.

Answer: Thank you for your observation. We have made the correction on line 118: “…the onset of the movement, maintaining or achieving proper…”

Comment: 4) line 123_Now line 128: “…relation between the level of performance stabilization level”. Repeated words.

Answer: Thank you for your observation. We removed the repeated word (line 128).

Comment: 5) Line 384_Now line 416: “The results demonstrated that…”. Demonstration seems more a result of logical reasoning, while “show” can be used as direct link to the data themselves. “The results showed that…” can be used instead.

Answer: Thank you for your observation. We changed the word “demonstrated” by “showed” on line 416.

Comment 6) Line 280 (as example): the term “main interaction effects” is frequently used in the Results section and is not appropriate. Traditionally, ANOVA has “main effects” (groups and blocks, in this study) and “interaction” (group by block, in this study).

Answer: Thanks for the correction. We have revised the description throughout the Results section.

References:

Burdet E, Tee KP, Mareels I, Milner TE, Chew CM, Franklin DW, et al. Stability and motor adaptation in human arm movements. Biol Cybern. 2006;94(1):20–32.

Campos CE, Matos CO, Silva LCC, Ribeiro PRA, Couto CR, Santos SP, Ugrinowitsch H. Kinematic Responses as a Control Strategy to Visual Occlusion. Computational Neurosciensce. p.250-262, 2022.

Corrêa UC, Benda RN, Oliveira DL, Ugrinowitsch H, Freudenheim A, Tani G. Different Faces of Variability in the Adaptative Process of Motor Skill Learning. Nonlinear Dynamics Psychology, and Life Science. v.19(4), p.465-487, 2015.

Couto CR, Leite CMF, Campos CE, Portes LL, Santos SP dos, Cruz MP, et al. Enhanced Performance Stabilization Increases Performance Variability in a Virtual Interception Task. Percept Mot Skills. 2021 Feb 1;128(1):546–59.

Craik KJW. The nature of explanation. Cambridge: Cambridge University Press, p. 126, 1943.

Cruz MP, Benda RN, Matos CO, Couto CR, Dutra LN, Costa CLA, Ugrinowitsch H. Combination of narrow and wide bandwidth knowledge of performance in the acquisition of a complex sports motor skills. Human Movement Science. v.95, p.103214, 2024.

Desmurget M, Grafton S. Forward modeling allows feedback control for fast reaching movements. Trends in Cognitive Sciences. v.4(11), p.423-431, 2000.

Ericsson K, Smith J. Prospects and limits of the empirical study of expertise. Em KA, Ericsson, J Smith (orgs.). Toward a general theory of expertise: Prospects and limits (pp. 1-38). Cambridge: Cambridge University Press, 1994.

Fonseca F de S, Benda RN, Profeta VL da S, Ugrinowitsch H. Extensive practice improves adaptation to unpredictable perturbations in a sequential coincident timing task. Neurosci Lett. 2012;517(2).

Loschiavo-Alvares FQ, Benda RN, Lage GM, Nicolato R, Ugrinowitsch H. Executive Functions and Motor Adaptation to Predictable and Unpredictable Perturbations. Percept Mot Skills. 2023;130(2).

McGarity-Shipley et al. (2023). Fast feedback responses to categorical sensorimotor errors that do not indicate error magnitude are optimized based on short- and long-term memory. The Journal of Neuroscience, 43, 8525-8535.

Moxley SE. Schema: The variability of practice hypothesis. Journal of Motor Behavior. v.11(1), p.65–70, 1979.

Santos SP, Benda RN, Couto CR, Campos CE, Andrade AGP, Lage GM, et al. The level of performance stabilization influences motor adaptation on an isometric force control task. PLoS One. v.12(10), 2017.

Schmidt RA. A schema theory of discrete motor skill learning. Psychological Review v.82(4), p.225–260, 1975.

Shea JB, Morgan RL. Contextual interference effects on the acquisition, retention, and transfer of a motor skill. Journal of Experimental Psychology: Human Learning and Memory. v.5(2), p.179–187, 1979.

Tresilian JR. Perceptual and cognitive processes in time-to-contact estimation: Analysis of prediction-motion and relative judgment tasks. Perception & Psychophysics. v.57, 1995.

Ugrinowitsch H, dos Santos-Naves SP, Carbinatto M V, Benda RN, Tani G. Motor skill adaptation depends on the level of learning. World Acad Sci Eng Technol [Internet]. v.77, p.956–960, 2011.

Ugrinowitsch H. Extensive Practice Improves Adaptation to Predictable Perturbations in a Sequential Coincidente Timing Task. American Journal of Life Sciences [Internet]. v.2(2), p.90, 2014.

Wolpert DM, Ghahramani Z, Jordan ML. An internal model for sensorimotor integration. Science. v.269, p.1880-1882, 1995.

Wolpert DM. Modular decomposition in visuomotor learning. Nature. v.386, p.392-395, 1997.

**REVIEWER 2**

Major comments:

We are grateful to the reviewers for their suggestions and the opportunity to improve the manuscript to make it clearer in this version. The comments made us think and write a lot about them, so some answers are quite long, and the whole file is nearly a new manuscript. Sorry about that. All changes in the manuscript are shown in blue.

Before answering the comments, it is important to explain that we changed PI by P_fast_ and PII by P_slow_ in the whole manuscript. We think it will make it easier for the reader to understand our design and Figures.

Major comments:

Introduction:

Comment 1) What is “performance stabilization” or “superstabilization”? It should be more clearly stated or defined from the outset. This is particularly relevant when consider the seemingly menial differences between the performance levels of these learning categories (i.e., 3 right trials for stabilization and 6 right trials for superstabilization; Ln. 192-195_Now lines 200-202).

Answer: This is our study's central point and we appreciate your comment. We have now worked on clarifying it (lines 70–79). Performance is considered stable when errors remain within a predefined range of accuracy defined as acceptable for a given task, and it is task-dependent. In our study, after conducting many pilot studies, we identified that volunteers were unlikely to repeat a performance with an error smaller than -5 cm to +5 cm from the center of the target more than three trials in a row over 500 attempts. Therefore, we adopted the criterion for this task as three consecutive trials with an error between -5 cm and +5 cm. This same criterion has been used in previous studies (Couto et al., 2021; Campos et al., 2022) for this task.

Specifically considering the two levels of stabilization, for performance to be stable (i.e., consistently reproduced within a small margin of error), there must be a spatiotemporal standardization of the movement, resulting in correct responses in consecutive trials, which allows us to consider that performance stabilization has been achieved (Burdet et al., 2006; Corrêa et al., 2015; Loschiavo-Alvares et al., 2023). This rationale has been applied in studies involving complex coincident-timing tasks, in which performance stabilization is reached when the error is equal to or less than 30 ms, and this level of performance is repeated for three consecutive trials (Fonseca et al., 2012; Ugrinowitsh et al., 2011; Campos et al., 2022; Loschiavo-Alvares et al., 2023). In an isometric force control task, we also ran pilot studies and found that participants could perform no more than four consecutive trials with an error smaller than 5% of the task goal (i.e., reach 40% of the maximum force). So, we adopted this performance criterion in a force control task (Santos et al., 2017). As described above, all these criteria were established following pilot studies to identify how volunteers perform according to the task.

Considering superstabilization requires another criterion, but pilot studies have shown that due to task constraints, performance accuracy does not increase indefinitely, as this would require a level of expertise. We would never be able to perform this study if we consider expertise as 10 years of practice or 10 thousand trials (Ericsson & Smith, 1994). Therefore, superstabilization was operationally defined as repeatedly maintaining stabilized performance. Again, based on pilot studies, we have adopted repeating the stabilization criteria for six blocks, as adopted in previous studies (Fonseca et al., 2012; Ugrinowitsh et al., 2011; Corrêa et al., 2015; Couto et al., 2021; Campos et al., 2022).

Previous studies (Ugrinowitsh et al., 2014 and Couto et al., 2021) have shown that, despite statistically equal accuracy during the learning phase, greater performance variability is identified when reaching superstabilization compared to those who practiced until stabilization. Still, most other studies that compared performance in stabilization with superstabilization did not find this behavior. This may happen because the differences can be found in control measures not in performance, and it depend on the characteristics of the task as well. However, this discussion about what happens in the phase prior to the insertion of perturbations was not the aim of the present study but rather to investigate behavior in the face of unpredictable perturbations.

Given the established criterion, we did not expect to find differences in performance during the first phase of the study (i.e., learning phase) but rather when participants had to face perturbations. Indeed, studies have shown that under such conditions, groups that practiced until reaching the superstabilization performance criterion (i.e., six blocks of three or four consecutive successful trials) could better deal with the perturbations than those that practiced only until reaching the performance criterion for what we named stabilization (Fonseca et al., 2012; Ugrinowitsh et al., 2014; Corrêa et al., 2015; Santos et al., 2017).

Comment 2) There should be mention of the plentiful work done around dealing with unexpected online perturbations in near-aiming tasks (with set targets) [Elliott et al., 2018, Crevecoeur, Scott & Cluff, 2019].

Answer: Thank you so much for the suggestion. We did not found Elliott et al. (2018), but we identified Crevecoeur et al. (2019) and found it very relevant. We have included the citation in the introduction (line 66) and the discussion (line 497).

Comment 3) While forming predictions surrounding “increased number of phase switches in the acceleration curve and a reduced time to reach peak velocity” (Ln. 132-136. Now lines 139-147), it remains unclear why this may be the case due to the absence of any prior explanation or rationale.

Answer: Between lines 120 and 124, we described the behavior of the speed and acceleration curves in unpredictable contexts and illustrated this with a description of the shape of curves showed in the study by Tresilian and Plooy (2006) on interception moving target tasks like in our study.

On line 139, where we presented our hypothesis, we replaced the term “switches” with “valleys” because “valleys” is the term we used on line 122 to describe the corrections/ adjustments observed in the acceleration curve.

Method:

Comment 4) As I understand it, there were a total of 129 trials with only 18 perturbation trials (x9 trials each positively [PI] and negatively [PII] accelerating) (Ln. 218-221_Now lines 233-239). Is this really enough or adequately representative of a corrective response?

Answer: In a previous study using a complex coincident timing task, Fonseca et al. (2012) adopted a design of an exposure phase with the same characteristics as in our study: the same number of control trials, the same number of perturbation trials, and the same arrangement of control and perturbation trials throughout the phase.

An analysis of the organization of the components of Fonseca et al. (2012)’ study showed that when facing unpredictable perturbations, participants changed the temporal organization of the task’s components compared with the organization in control trials, and SSG used a different strategy to coincide the movement with the visual stimulus. This result indicates that the Exposure phase structure is sufficient for identifying changes in the movement control aiming to perform a correct trial.

In our study of a moving target interception task, we identified corrections made in response to perturbations through a series of previous pilot tests. Furthermore, Figures 3a and 4a show the number of corrections made by each group, demonstrating that, yes, the arrangement of the Exposure phase is sufficient to elicit or adequately represent corrective responses.

However, it is important to clarify that we adopted the definition corrections in moving target interception tasks based on the study by Tresilian and Plooy (2006), which means changes on the acceleration curve.

Comment 5) Why limit the study or analysis of “exposition” to CE and tPV%? Why not N-corr?

Answer: We appreciate your question, as it drew our attention to the importance of mentioning this measure at the beginning of the manuscript.

In this manuscript, we investigated the effects of two stabilization levels on performance and control mechanisms. The CE is our performance measure, and we adopted it because it indicates direction on performance during intercepting moving targets (early or later in relation to the target). Considering the mechanisms of control, we can analyze whether the movement was preprogrammed or controlled by online feedback. The peak velocity is a common measure of preprogram because the proximity of the peak velocity to the movement time minimizes the possibility of corrections, which indicates high participation of the preprogram control mechanism.

On the opposite direction, the peak velocity early in relation to the movement time indicates time to make corrections, indicating participation of the online feedback control mechanism. Based on this explanation, we adopted tPV%, aiming to have higher control of the relation between time to peak velocity and movement time since movement time is variable.

The N-cor was adopted to evaluate the number of corrections, which indicates the use of online feedback control mechanism. So, these three measures were enough to answer our question. However, considering the importance of N-cor in the exposition phase, your comment made us realize the need to explicitly state in abstract, that the adjustments we refer to in the title and throughout the manuscript were identified based on the N-cor analysis (lines 43 and 44).

For example, it is possible to find the descriptions of the N-cor results, as illustrated in Figures 3a and 4a, and located as follows: P1_fast_ (lines 291 and 292), P2_fast_ (lines 304 and 305), P3_fast_ (lines 320 and 321); P1_slow_ (lines 343 and 344), P2 _slow_ (lines 356 and 357), P3 _slow_ (lines 377 and 378).

Comment 6) If “exposition” is of interest, then why not incorporate it as a factor in a more complex omnibus ANOVA (e.g., group x exposition x trial). As it stands, the inferential stats are somewhat fragmented and potentially limit any over-arching conclusions that could be made.

Answer: Thank you for the suggestion. This analysis would indeed be very interesting for identifying changes/learning throughout the exposure phase, but the proposed analysis answers a different question. We introduced the perturbations primarily to identify changes in motor control as participants tried to overcome them.

The suggested analysis is very interesting and allows us to answer questions related to motor learning while facing perturbations. We got excited about this idea, so we proposed to run the proposed analysis in a similar study that we are conducting in our group.

Results:

Comment 7) Interactions between group and block are identified (Ln. 289_Now line 310 and Ln. 303_Now line 326), although the pairwise comparisons reported in the text does do not shed any further light on the direction of these interactions. Along these lines, some of pairwise comparisons that are drawn appear inconsistent with some making comparisons within- (Ln. 290-291_Now lines 311-312) and others between-measures (Ln. 336-337_Now lines 360-361).

Answer: You are right. Your comment led us to discuss how we described the interactions and, we recognize the importance of specifying the direction of interactions using the terms “later” or “earlier”, within parentheses, in the post-hoc description. So, we ensure that all interactions are explicitly indicated in the post-hoc analysis. On lines 312, 314, 327, 329, 369-370, 386-387, 388, we specified that the difference was between groups SG and SSG.

Comment 8) Along these lines, the symbol () indicating statistically significant pairwise differences in both figures 3 and 4 look like they could be highlighting differences between-measures (i.e., SSG vs. SG), although the figure captions allude to mere “interactions differences”. If comparisons are drawn within- and between-measures, then use different symbols (e.g., [] between, [†] within) in different locations (e.g., adjacent to data points for between and adjacent to lines for within) accordingly.

Answer: Your comment shows how careful you were in analyzing our manuscript. Thank you very much.

The figures illustrate the differences between the two groups (SG and SSG). However, these differences were indicated only in specific blocks, specifically in those blocks where the ANOVA showed interactions (tPV% and CE measures). Therefore, in the graphs, we used only one symbol (*) to indicate differences between groups in specific blocks. In other words, the symbol (*) was inserted only in blocks where post-hoc analysis identified significant differences between groups.

Comment 9) In the multiple reports for CE, the direction of effects can sometimes conflate increases/decreases with error away from 0 (i.e., perfection). Rather than being too literal in describing the direction on the scale, perhaps it would be more informative to indicate the implications on error (e.g., “more negative/positive error”, “more susceptible to the negatively accelerating target”). This is not to say that direction is not important to observe because it indicates the impact of the perturbation (e.g., positively accelerating target should delay the response and add (+) to the CE score), although the nature of responses in terms of distance from 0 should be highlighted more often.

Answer: Your observation allowed us to consider a way to provide a better perspective on the groups’ performance in response to perturbations.

To indicate the nature of the response in relation to zero, which represents the center of the target, we inserted the terms “later”, “earlier”, and “closest to zero” in the following lines: 297, 299, 311, 313, 316, 326, 328, 330-331, 349, 351, 367, 369, 370-371, 373, 386, 387, 389, 391 and 393. Thank you.

Minor comments:

Method:

Comment 1) Why an effect size of 0.3 (Ln. 145_Now line 149)? Based on what prior effects and related studies?

Answer: Yes. We used Cohen’s high effect size (η_p_² above 0.14) and the mean η_p_² values found in the analyses of motor control variables, biceps and triceps muscle activity, and biceps/triceps co-activation in response to unpredictable perturbations in the study by Santos et al. (2017), which manipulated the same variable as our study (performance stabilization levels). The η_p_² values reported by Santos et al. (2017) were: η_p_² = 0.37 (biceps); η_p_² = 0.43 (triceps); η_p_² = 0.23 (biceps/triceps co-activation).

Comment 2) Does “reability” mean to be “reliability” (Ln. 146_Now line 151)??

Answer: Yes, that's right. Thank you for the observation. It has been fixed on line 151.

Comment 3) Given the disparity between the aiming movement surface and projector display (Ln. 156-160_Now line 161-167), what was the movement gain or cursor-to-pen mapping?

Answer: Thank you for your question because it warned us about the importance of taking this more clearly in the manuscript. Then, we made an insertion on line 184.

The gain was in movement control, resulting in the pen’s movement, which covered “exactly” the same distance both the table and the virtual environment. During the task, the participant observed the target’s movement, and within 200–250 ms, they learned to control this movement time to align the effector’s movement with the target’s arrival in the interception zone.

Comment: 4) The mention of “target velocity” and “time window” is somewhat lost on me (Ln. 186_Now line 193 and Ln. 222-223_Now lines 238-239). What is the “time window” in reference to?

Answer: You are right; we could have specified in the manuscript which variables were considered for the time window. We have added this information on lines 192 and 193.

The object is in the strike zone for a period termed the time window. This window in time quantifies the extrinsic constraint on the temporal accuracy of a successful strike. The time window can be manipulated in one or more of three ways: by changing effector size, target size, or target speed. In our study, we manipulated the target speed and showed each time window in the lines 193and 239.

The following equation calculates the time window:

*Target’s length + effector width / target’s speed*

(Tresilian, 1999, 2005; Tresilian and Lonergan, 2002)

Comment 5) I take it that a 145 cm/s target velocity with a 210 cm amplitude (until reaching the intercept zone) would mean the target moves for over 1 sec (1.45 secs to be precise). There in with a 200-250 ms MT, then participants should have initiated their movements some time way after target motion initiation. Please clarify.

Answer: Your question made us realize the importance of specifying the target's displacement distance from its appearance to the interception zone, and your comment represents what happened in the task. Therefore, we included this information in lines 179 and 180.

Below, we provide more details about the displacement:

In the control trials, the target moved at 145 cm/s over 213 cm in a virtual rail, resulting in a displacement time of 1.468 seconds. Therefore, 1.468 seconds also corresponded to the participant's viewing time. Participants initiated their movements after the target started moving. They had sufficient time (1.468 sec) to identify the target’s characteristics before interception and plan the movement considering the time constraint of 200-250 ms. The target displacement time, or participant viewing time, was determined through pilot studies based on previous research using a similar task, such as Tresilian and Houseman (2005) and Tresilian et al. (2009).

In addition, we showed the time window to demonstrate that the participant had sufficient time to identify the target information, execute the movement within the specified time (200-250 ms), and attempt to hit the target.

Comment 6) With a 200-250 ms MT and 27.7 cm movement amplitude, then the intended limb velocity was around 138 cm/s. Please clarify.

Answer: Thank you! This is an important point. We had not considered including this information; however, your comment made us realize the importance of providing this detail (lines 218-223). To maintain a movement time between 200 and 250 ms while the effector moves over a distance of 27.7 cm, the limb's movement speed should range between 110.8 and 135 cm/s.

Comment 7) Figure 2 – Having a “blue target” and “red effector” seems odd to me, particularly when they were colored yellow and green, respectively (like in Figure 1).

Answer: Thank you for your comment; it was odd. We have modified the target's color (yellow) and effector in Figure 2.

Comment 8) There does not seem to be any prior mention of “exposition” (Ln. 245)? Does it need to be “exposure”?

Answer: Thank you for your comment. Yes, it needs to be exposure. We have changed “exposition” to “exposure" on lines 262, 268, 269, 270, 280, 424.

Comment 9) As I understand it, there were x9 trials each positively (PI) and negatively (PII) accelerating trials (Ln. 219-220_Now lines 235-236). With 3 “blocks” of “exposition” (Ln. 251-253_Now lines 274-276), then would that mean there were x3 trials devoted to each level of block (e.g., Pre PI2 = x3 trials)?

Answer: Yes, each block (Pre-perturbation, Perturbation and Post-perturbation) consisted of three trials. On lines 271-273, we inserted information concerning the specific number of trials in each block.

References:

Burdet E, Tee KP, Mareels I, Milner TE, Chew CM, Franklin DW, et al. Stability and motor adaptation in human arm movements. Biol Cybern. 2006;94(1):20–32.

Campos CE, Matos CO, Silva LCC, Ribeiro PRA, Couto CR, Santos SP, Ugrinowitsch H. Kinematic Responses as a Control Strategy to Visual Occlusion. Computational Neurosciensce. p.250-262, 2022.

Cohen J. Statistical power analysis for the behavioural sciences. Hillside. NJ: Lawrence Earlbaum Associates. 1988.

Corrêa UC, Benda RN, Oliveira DL, Ugrinowitsch H, Freudenheim A, Tani G. Different Faces of Variability in the Adaptative Process of Motor Skill Learning. Nonlinear Dynamics Psychology, and Life Science. v.19(4), p.465-487, 2015.

Couto CR, Leite CMF, Campos CE, Portes LL, Santos SP dos, Cruz MP, et al. Enhanced Performance Stabilization Increases Performance Variability in a Virtual Interception Task. Percept Mot Skills. 2021 Feb 1;128(1):546–59.

Crevecoeur F., Scott SH., Cluff, T. Robust control in human reaching movements: a model-free strategy to compensate for unpredictable disturbances. Journal of Neurophysiology, 39(41), 8135-8148, 2019.

Ericsson K, Smith J. Prospects and limits of the empirical study of expertise. Em KA, Ericsson, J Smith (orgs.). Toward a general theory of expertise: Prospects and limits (pp. 1-38). Cambridge: Cambridge University Press, 1994.

Fonseca F de S, Benda RN, Profeta VL da S, Ugrinowitsch H. Extensive practice improves adaptation to unpredictable perturbations in a sequential coincident timing task. Neurosci Lett. 2012;517(2).

Loschiavo-Alvares FQ, Benda RN, Lage GM, Nicolato R, Ugrinowitsch H. Executive Functions and Motor Adaptation to Predictable and Unpredictable Perturbations. Percept Mot Skills. v.130(2), 2023.

Santos SP, Benda RN, Couto CR, Campos CE, Andrade AGP, Lage GM, et al. The level of performance stabilization influences motor adaptation on an isometric force control task. PLoS One. v.12(10), 2017.

Tresilian JR. Visually timed action: time-out for ‘tau’? Trnds in Cognitive Sciences. v.3(8), p.301-310, 1999.

Tresilian JR, LONERGAN A. Intercepting a moving target: effects of temporal precision constraints and movement amplitude. Experimental Brain Research, v. 142, p. 193-207, 2002.

Tresilian JR. Hitting a moving target: perception and action in the timing of rapid interceptions. Perception & Psychophysics. v. 67, n. 1, p. 129 – 149, 2005.

Tresilian JR, Plooy A. Systematic changes in the duration and precision of interception in response to variation of amplitude and effector size. Experimental Brain Research. v.421–435, 2006.

Ugrinowitsch H, dos Santos-Naves SP, Carbinatto M V, Benda RN, Tani G. Motor skill adaptation depends on the level of learning. World Acad Sci Eng Technol [Internet]. v.77, p.956–60, 2011.

Ugrinowitsch H. Extensive Practice Improves Adaptation to Predictable Perturbations in a Sequential Coincidente Timing Task. American Journal of Life Sciences [Internet]. 2014;2(2):90.
