# Supplementary material for: Practice beyond performance stabilization increases the use of online adjustments to unpredictable perturbations in an interceptive task
Source: PLoS One. 2025 Nov 5;20(11):e0313665. doi: 10.1371/journal.pone.0313665 (PMC12588517; doi:10.1371/journal.pone.0313665)
Supplement: Attachment — Submitted filename: Response_to_reviewers_auresp_2.docx [file pone.0313665.s004.docx]

Response to Reviewer 2

We sincerely thank the reviewers again for their news insightful comments and constructive suggestions, which have significantly contributed to enhancing the clarity and overall quality of the manuscript. All modifications made to the manuscript are indicated in blue.

**Major comments**

**Introduction:**

**1)** While stabilization has been operationally defined within the manuscript, I find the explanation offered in the response letter to be more informative. Perhaps include some of this content, including details of empirical findings (e.g., Campos et al., 2022), in order to better envisage the state of (super-)stabilization.

*We completely agreed that including empirical details would facilitate a better understanding of the state of (super-)stabilization. So, we incorporated this information between lines 68 and 114 (blue lines).*

**Method**

**2)** I remain unconvinced by the failure to incorporate “exposure” into the analysis. It has been explained within the response letter that changes/learning were not of primarily concern when it came to the perturbation trials, although it begs the question: why consider “exposure” in the first place including the separation of perturbation trial blocks (i.e., x3 trials per block)?

*We considered exposure in the first place because our aim was to analyze the control and performance of two levels of performance stabilization in the face of unpredictable perturbations. We did not consider the first phase (learning phase) because we took as a starting point what other studies have already shown: the stabilization and superstabilization groups already end the learning phase differently, with different skills. The difference between the two groups has been shown in studies such as, for example, Ugrinowitsch et al. (2011); Fonseca et al. (2012); Ugrinowitsch et al. (2014); Santos et al. (2017); Couto et al. (2021). Therefore, we assumed that the groups are different and inserted them into a phase with 18 disturbances, nine fast and nine slow.*

*In the exposure phase, we chose to analyze our dependent variables in three blocks of three trials at the pre- and post-perturbation moments, and not in just one block with nine trials, because the first organization allows us to glimpse the effects of the independent variable from the beginning to the end of the exposure phase. More specifically, we can glimpse at different moments (which we understand as beginning, middle and end of exposure) throughout the 129 trials, when each group begins to modify their control strategies and at what moment these modifications become effective, that is, they influence performance in a positive way in the face of the perturbations.*

*If we had analyzed the trials in just one block, we would only be able to show that the groups deal differently with the perturbations and also that they are affected differently by them after they are removed (moment Post-P). However, this second analysis did not allow us to see, for example, that despite the superiority of the superstabilization group, at the beginning of the exposure, when the first PII were inserted, this group showed a control strategy (tPV%) and performance (CE) similar to the stabilization group. However, in the middle and end of the exposure, the superstabilization group showed an effective control strategy to maintain performance, which the stabilization group was not able to.*

*The figure below illustrates the performance of the stabilization and superstabilization groups in just one block with the nine PII. If we had used this analysis, we could interpret that the superstabilization group had already started the exposure phase superior to the stabilization group, which the analysis in blocks with three trials shows is not true.*

**Results**

**3)** While the direction of effects has been made much clearer, the failure to decompose interactions within the text still remains. Instead, I’m having to solely observe the between-group differences purported in figures 3 and 4 (via symbols) because the text mostly describes common differences between blocks within SG and SSG groups (synonymous with a block main effect) (Ln. 312-315, 327-330). When it comes to the interaction, I would advise focusing the report on the differences highlighted within the fore mentioned figures (e.g., CE in fast speed perturbation: SSG < SG for P, SSG > SG for Post).

*We appreciate the suggestion and rewrite the results of the interactions in the lines that there are in blue color between lines 351 and 435.*

**Minor comments**

**Introduction**

**4)** I may have misquoted the year of citation for Elliott et al. Here is the reference:
Elliott et al. (2017). The multiple process model of goal-directed reaching revisited. Neuroscience & Biobehavioral Reviews, 72, 95-110.

*Thak you by the reference.*

**Method**

**5)** With regard the physical-to-virtual movement gain, there is mention of movement on the table and display being the same, but that can’t be possible. The movement on the table was only ~27 cm, while it was projected onto a large projector nearing a 2-m vertical amplitude (i.e., movement on the display will have gone visually further and faster than the limb in reality).

*Yes, the vertical projector had a total length of 228 cm, but we configured the virtual effector image to measure only 27 cm, as illustrated in the figure 1. We have made adjustments to the size of the effector rail in figure 1 in a way that it is more proportional to the actual size. Thank you for your attention with our manuscript.*

**6)** The following description does not seem to compute: “The velocity at which the target moved was 145 cm/s. A 4 × 6 cm target moving at 145 cm/s and a 2 × 4 cm effector resulted in a 68,96 ms time window” (Ln. 192-193). Is this 6,896 ms (6.896 secs)? If the target is travelling 213 cm at 145 cm/s, then it thought to be nearer ~1.5 secs of travel time.

*We understand your question, and we realized that the definition of time window was not well described in line 232, which caused this doubt. We improved the writing (lines 233 and 234) to make it clearer. The time window does not refer to the travel time, but rather to the time in which the target is within the strike zone and can be contacted by the effector.*

**7)** The following description could be more clearly worded: “After 10 min of reaching the performance criterion started the Exposure phase” (Ln. 233-234).

*Thank you by the suggestion. We appreciate it and incorporated it in lines 273 and 274.*

**8)** While the rationale for only 9 trials per category of perturbation is robust within the response letter, we’re none-the-wiser when it comes to reading solely the manuscript. Some of the logic explained (including Fonseca et al. citation) should be highlight here (Ln. 236).

*Thank you for the suggestion. We appreciate it and made the change between the lines 277 and 281.*

**9)** It states “The time window of Pfast was 50 ms, and the time window of Pslow was 111 ms” (Ln. 239), although if the onset of the target velocity perturbation was contingent upon the onset of the interceptive movement (with varying time to initiate and velocity of movement), then should the “time window” not between trials.

*We understand that the definition of time window was not well described in line 224, which caused this doubt. Considering the corrected form in the writing (lines 233 and 234), the change in the target's speed will cause a change in the possible time of contact between the effector and the target in the strike zone (time window). Therefore, we describe the changes in the time windows caused by the perturbations.*

**10)** Upon second reading of the calculus of error (Ln. 206-207, 254-255), I’m unsure exactly about how it was really done. The description in Ln. 206-207 would make me think it was the distance between the target (yellow) and effector (green) at the moment of the former reaching the “strike zone”. However, viewing Figure 3 would make me think it was all about the distance between the target and “strike zone” at the moment that the effector reached the “strike zone”. Please explain and make clear the implication of direction (i.e., negatively signed scores indicate too slow an interceptive movement, while positively signed scores indicate a pre-exempted response).

*We actually understood that the description of how the constant error was obtained was not clear. Moreover, your understanding from the figure is correct. We insert the information about the constant error in lines 297-299.*

**11)** The explanation offered for the “Pre” and “Post” trials is not entirely clear (Ln. 264-268). For example, the following statement on “The Pre trials are the reference to identify what happens with the performance and with the motor control when perturbations are inserted” reads like it is the perturbation trials themselves (thus bearing no distinction from “P trials”).

*We improved the wording to make it clearer between lines 308 and 310. Thank you for the suggestion.*

**12)** The detail behind the perturbation blocks also gets somewhat muddled (Ln. 271-273). There has to be an easier way to explain or simplify this.

*Thank you for the observation. We a change the write between the lines 310 and 315; 319 and 321.*
