# Supplementary material for: Practice beyond performance stabilization increases the use of online adjustments to unpredictable perturbations in an interceptive task
Source: PLoS One. 2025 Nov 5;20(11):e0313665. doi: 10.1371/journal.pone.0313665 (PMC12588517; doi:10.1371/journal.pone.0313665)
Supplement: Attachment — Submitted filename: Response_to_Reviewers_auresp_3.docx [file pone.0313665.s005.docx]

**Response to the Editor**

*We sincerely thank the Editor and Reviewer 2 for their attention to our manuscript and for the opportunity to receive important feedback to improve the quality of our final product.*

*We reorganized the variable names to make them consistent with the data analyses, as Reviewer 2 identified coherence issues. All modifications made to the manuscript are indicated in green and have been incorporated into the graphs.*

*In the Measures and Data Analysis section, we modified the order of the dependent variables description (lines 296–305) to maintain consistency with the order of presentation in the Results, which was: number of corrections, relative time to peak velocity, and constant error. In the previous version of the manuscript, these variables had been presented in the following order: constant error, time to peak velocity, and number of corrections.*

**Response to Reviewer 2**

*We sincerely thank the reviewer 2 once again for insightful comments and constructive suggestions. We feel privileged to receive such valuable feedback, which has significantly contributed to improving the clarity of our manuscript.*

*All modifications made to the manuscript are indicated in green.*

Major Comments:

**1)** The explanation of the trials does not entirely make sense, and what’s more, it may not even help matters. Either remove or elaborate (Ln. 310-315).

*Thank you for the suggestion. We removed it, and the paragraph is clearer now (see topic Measures and data analysis).*

**2)** The difficulty in understanding may be the distinction between what is effectively x3 levels of exposure (pre, intermediate, post-) and x3 levels of block (trials 1-3, 4-6, 7-9). That is, there appears a confluence in the explanation for each of these factors (Ln. 316-321). Perhaps more definitively distinguish each of these (e.g., …exposure pertained to the immediately before (Pre), during (P) and after (Post) a perturbation, which was further brokered into block 1 (trials 1-3), block 2 (trials 4-6) and block 3 (trials 7-9).

***Thank you for your observation. You are correct; there was indeed some overlap in the explanation regarding blocks and moments. We reorganized the variable names to ensure they are consistent with data analyses.***

***First, we would like to clarify that “exposure” is not the moment with the Perturbation. The “exposure” refers to the entire experimental phase, comprising 129 trials. The exposure phase includes111 control trials, 9 fast perturbation trials (P_fast_), and 9 slow perturbation trials (P_slow_). We attempted to make this clear in lines 274 and 275 of the revised manuscript.***

***Regarding our confluence of*** *the factors, to improve clarity, we revised the text between lines 312 and 337, as well as in the description of the results (lines 346-451).* ***Each type of perturbation (i.e., fast and slow) was organized into three “moments”: Early (trials 1-3), Intermediate (trials 4-6), and Later (trials 7-9). Each “moment” was divided into three blocks — Pre, P (perturbation), and Post — and each block represents the average of three trials, which means we organized each perturbation trial with its respective surrounding control trials (Pre and Post).*** *This organization was applied separately to fast and slow perturbations.*

**3)** The nomenclature used to define exposure and block is not exactly intuitive or easy to follow (i.e., “Block 1 with Pre, P1fast and Post, b) Block 2 with Pre, P2fast and Post…”) (Ln. 321-323). How about something like the following instead?: Pre1-3fast/slow, P1-3fast/slow, Post1-3fast/slow.

*This question is closely linked to Question 2, and we likewise acknowledge that the original description may have led to confusion. We believe that the changes made in lines 311–338 and 347, 360, 378,402, 415 and 436 clarify the nomenclature problem.*

**4)** Fore mentioned confusion is further compounded by the description of the two-way ANOVA where it appears block (1-3) was featured as a factor, but in reality it was exposure (Pre, P, Post) (Ln. 327-329).

*Your observation is correct. As we cited before, the confusion stemmed from an imprecise description of the relationship between blocks and moments. We reorganized the whole manuscript based on the answer to comment #2. Summarizing*

- *Early Moment: trials 1–3 with perturbation, along with their corresponding Pre and Post trials;*
- *Intermediate Moment: trials 4–6 with perturbation and their respective Pre and Post trials;*
- *Later Moment: trials 7–9 with perturbation and their corresponding Pre and Post trials.*

*Two-way ANOVA was conducted between the two groups to compare the following variables: N-Cor, tPV%, and CE across the three blocks (Pre, P, and Post), separated by moment (Early, Intermediate and Later) and type of perturbation, P_slow_ and P_fast_.*

*We have modified the text between lines 336 and 338 to improve clarity and address the reviewer’s comment.*

**5)** (Unless I’m missing something completely here!) The same problem applies for the description of the inferential statistics (i.e., there is a constant reference to ‘blocks’ when I think it should be ‘exposure’, while blocks [1-3] were assessed separately in different sub-sections of the Results) (Pg. 14-18).

*You are correct, the original description was unclear. We believe we have addressed this issue with the revised explanation provided between lines 313 and 338, as well as with the updated symbols of figures 3 and 4. The statistical description is now correct and consistent with the definitions of blocks and moments presented in that section.*

**6)** While pairwise difference have been more explicitly incorporated, they fail to decompose the group x block (i.e., exposure) interaction (e.g., “Post hoc analysis revealed that the same pattern was observed for the GS (p < 0.05)”). That is, the inferential stats report a similar pattern of influence of exposure within SSG and SG; thus, there would seem no difference between groups as a function of exposure. Meanwhile, there are asterisks within Figure 3 and 4 alluding to so-called “Interaction differences”. Due to this uncertainly we cannot really comprehend what has really been found here, and thus we cannot conclude anything concrete.

*The same behavioral pattern was observed for SG and SSG only in relation to the behavioral tendency towards control and perturbation trials. For example, when the perturbation was introduced, the CE of both groups increased compared to the control trials (Pre block), in both P_slow_ and P_fast_ conditions. Despite the increase, for example, in the Last Moment of P_Slow_, the GSS showed a lower constant error than SG in the face of perturbations. Subsequently, when the perturbation was removed, the constant error of both groups decreased in the control trials (Post) compared to the perturbation trials (P) in both fast and slow perturbations. This description elucidates the same behavioral pattern (increase (in P) and decrease (in Post) of error) for SG and SSG, but with SG being superior only on Intermediate and Last Moments of Slow Perturbation. This is because this group, despite having an increased error in P compared to the Pre, presented a lower constant error than SG when faced with the Perturbation slow (P4-6 _slow_ and P7-9_slow_).*

*More specifically, in the decomposition of interaction groups x blocks, a similar pattern of behavior between SG and SSG was seen in the Intermediate and Last moments of the fast perturbation, but without superiority of SG, as shown below and described in detail between lines 367-373 and 383-389 of the manuscript.*

1) Intermediate Moment: Pre x P4-6_fast_ x Post

*Dependent variable: Constant Error (CE)*

*Description: In relation to control trials (Pre), the CE increased in both groups when the perturbation was inserted (P4-6_fast_), then when the perturbation was withdrawn (Post), the CE decreased in relation to P4-6_fast_. Specifically, in this moment (Intermediate Moment), for fast perturbations, the SSG is not superior to the SG. Therefore, both groups showed a similar behavior pattern.*

2) Last Moment: Pre x P7-9_fast_ x Post

*Dependent variable: Constante Error (CE)*

*Description: In relationship to control trials (Pre), the CE increased in both groups when the perturbation was inserted (P7-9_fast_), then when the perturbation was withdrawn (Post), the CE decreased in relation to P7-9_fast_. Specifically in this moment (Intermediate Moment), to fast perturbation, the SSG it is not superior to the SG. Therefore, both groups showed a similar behavior pattern.*

*Regarding the slow perturbation, although SG and SSG showed a similar pattern of behavior at the Intermediate and Last moments, SSG was superior to SG. The behavior patterns are presented below and described in detail between lines 428–431 and 444–457 of the manuscript.*

1) Intermediate Moment: Pre x P4-6_slow_ x Post

*Dependent variable: Constante Error (CE)*

*Description: In relationship to control trials (Pre), the CE increased in both groups when the perturbation was inserted (P4-6_slow_), then when the perturbation was withdrawn (Post), the CE decreased in relation to P trials. It is important to highlight that in this moment (Intermediate Moment), SSG showed lower CE than SG in P4-6_slow_, i.e., when P_slow_ was inserted. Furthermore, the SSG superiority can be observed by comparing the CE in the perturbation block with the previous control trials (Pre). This comparison shows that the SSG, unlike the SG, was able to maintain its performance (CE) from the previous control trials (Pre) even after the perturbation was inserted. In contrast, SG increased the CE in response to the perturbation when compared to its performance in the previous control trials (Pre).*

2) Intermediate Moment: Pre x P7-9_slow_ x Post

*Dependent variable: Constante Error (CE)*

*Description: In relationship to previous control trials (Pre), the CE increased to both groups when the perturbation was inserted (P7-9_slow_), then when the perturbation was withdrawn (Post), the CE decreased in relationship P7-9_slow_. It is important highlight that in this moment (Intermediate Moment), the SSG showed lower CE than SG in P7-9_slow_, i.e. when P_slow_ was inserted. Furthermore, SSG superiority can be observed by comparing the CE in the perturbation block with the block of previous control trials (Pre). This comparison shows that the SSG, unlike the SG, was able to maintain its performance (CE) from the previous control trials (Pre) even after the perturbation was inserted. In contrast, SG increased the CE in response to the perturbation when compared to its performance in the block of previous control trials (Pre). Finally, in this moment (Last Moment), it was found difference between groups. The post-hoc showed that SSG had lower CE than SG.*

*The superiority of SSG's performance can be understood from the analyses of N-cor and tPV%. The results of these motor control variables showed that, at the "Intermediate" (P4-6 slow) and "Last" (P7-9 slow) moments, when perturbations were introduced, SSG presented a lower tPV% and performed more corrections than SG. These results indicate that, when the perturbation is slow, the group that achieves superstabilization of performance is more competent in using the strategy of reducing the time to peak velocity, which allows make corrections in the Last portions of the movement. Furthermore, SSG performed a higher number of corrections than SG, which were also more effective, during P_slow_. These results allow for the maintenance of the previous performance demonstrated Pre trials, and also have a superior performance than SG, which also performed corrections, but not as effectively as SSG.*

*We updated the symbols representing the results of the interactions and updated the captions in lines 397-398 and 460-461.*

Minor comments

**7)** “Consecutives” should be “consecutive” (i.e., no “s”) (Ln. 92).

*Thank you for the observation. We made the correction on line 92.*

**8)** “Internal Models” is capitalised either in error or for some unknown reason.

*Thank you by observation. We made the correction on line 118.*

**9)** Excessively long sentence with some missing connecting words and/or punctuation (Ln. 115-120).

*Thank you for the observation. We inserted a full stop on line 118.*

**10)** “GS” typos instead of “SG” (e.g., Ln. 419).

*Thank you for the observation. We made the correction on lines 371, 387, 430 and 449.*

**11)** Tendency to mix up the past and present terms for ‘withdraw’ (e.g., “withdrawal” instead of “withdrawn”).

*Thank you by observation. We made the correction on lines 354, 369, 372, 386, 388, 409, 423, 428, 448 and 452.*
