# Supplementary material for: Practice beyond performance stabilization increases the use of online adjustments to unpredictable perturbations in an interceptive task
Source: PLoS One. 2025 Nov 5;20(11):e0313665. doi: 10.1371/journal.pone.0313665 (PMC12588517; doi:10.1371/journal.pone.0313665)
Supplement: Attachment — Submitted filename: Response_to_Reviewers_auresp_4.docx [file pone.0313665.s006.docx]

*We sincerely thank Reviewer 2 for the attention to our manuscript. All modifications made to the manuscript are indicated in green.*

Minor comments:

1) The modified description of the analysis could be made clearer and more explicit (Ln. 335-337). Perhaps the following: “The CE and tPV% measures from each of the exposure phase moments (Early, Intermediate and Later) and perturbations (fast, slow) were analyzed using a two-way mixed-design ANOVA consisting of group (SG and SSG) and block (Pre, P, Post) factors.”

*We have made the modification as suggested between lines 335 and 337.*

2) Post hoc analysis was unable to decouple the interaction between group and block for the following: Intermediate moment: Pre-P x P4-6fast x Post P (Ln. 366-372), Later moment: Pre-P x P7-9fast x Post P (Ln. 382-388). This should be made clearer within the text report (along similar lines to that reported by the authors in the previous ‘response to comments’ letter).

*We have rewritten it as suggested between lines 369–375 and 387–393.*

3) Potential misreport in the post hoc that decouples the interaction (Ln. 430): “…the SSG showed lower (closest to zero) CE than SG in P4-6slow and similar CE in Pre and P4-6slow (p < 0.05).”

*Indeed, P4-6slow was a misreport. Regarding CE, the similarity between groups at the intermediate moment occurred in the Pre and Post perturbation phases, but not during the perturbation (P4-6slow). We have made the correction in line 435.*
